# Innovative Three-Dimensional Microscopic Analysis of Uremic Growth Plate Discloses Alterations in the Process of Chondrocyte Hypertrophy: Effects of Growth Hormone Treatment

**DOI:** 10.3390/ijms21124519

**Published:** 2020-06-25

**Authors:** Ángela Fernández-Iglesias, Rocío Fuente, Helena Gil-Peña, Laura Alonso-Durán, María García-Bengoa, Fernando Santos, José Manuel López

**Affiliations:** 1 Division of Pediatrics, Department of Medicine, Faculty of Medicine, University of Oviedo, CP 33006 Oviedo, Asturias, Spain; angelafiglesias@gmail.com (A.F.-I.); rociofuenteperez@gmail.com (R.F.); hgilpena@gmail.com (H.G.-P.); laurita.alonso86@gmail.com (L.A.-D.); mariagbengoa18@gmail.com (M.G.-B.); jmlopez@uniovi.es (J.M.L.); 2 Instituto de Investigación sanitaria del Principado de Asturias (ISPA), 33012 Oviedo, Spain; 3 Department of Pediatrics, Hospital Universitario Central de Asturias (HUCA), 33013 Oviedo, Asturias, Spain; 4 Department of Morphology and Cellular Biology, Faculty of Medicine, University of Oviedo, CP 33006 Oviedo, Asturias, Spain

**Keywords:** chronic kidney disease, CKD, uremia, chondrocyte, hypertrophy, GH

## Abstract

Chronic kidney disease (CKD) alters the morphology and function of the growth plate (GP) of long bones by disturbing chondrocyte maturation. GP chondrocytes were analyzed in growth-retarded young rats with CKD induced by adenine intake (AD), control rats fed ad libitum (C) or pair-fed with the AD group (PF), and CKD rats treated with growth hormone (ADGH). In order to study the alterations in the process of GP maturation, we applied a procedure recently described by our group to obtain high-quality three-dimensional images of whole chondrocytes that can be used to analyze quantitative parameters like cytoplasm density, cell volume, and shape. The final chondrocyte volume was found to be decreased in AD rats, but GH treatment was able to normalize it. The pattern of variation in the cell cytoplasm density suggests that uremia could be causing a delay to the beginning of the chondrocyte hypertrophy process. Growth hormone treatment appears to be able to compensate for this disturbance by triggering an early chondrocyte enlargement that may be mediated by Nkcc1 action, an important membrane cotransporter in the GP chondrocyte enlargement.

## 1. Introduction

Growth impairment remains a major complication in pediatric patients with chronic kidney disease (CKD) and only 30% of adults with childhood onset CKD reach a normal final height [1,2]. Several factors play a causal role in this growth retardation, including small infant size at birth, metabolic acidosis, salt and water deficits, anorexia, malnutrition and cachexia, anemia, and resistance to anabolic hormones such as growth hormone (GH), insulin-like growth factor 1 (IGF1), and sexual hormones.

Longitudinal growth is the product of an elaborate cascade of events, many of them taking place in the cartilaginous center of long bones, the epiphyseal growth plate (GP) [3]. Chondrocytes within the GP elongate the bone by the proliferation, progression, hypertrophy, and synthesis of the extracellular matrix [4]. The columnar or proliferative chondrocytes exit the cell cycle and begin to increase their cell volume and change their cell shape to form prehypertrophic and hypertrophic chondrocytes. The hypertrophic cells mineralize their extracellular matrix and either they are degraded by osteoclasts and replaced by invading osteoblasts or they transdifferentiate into bone-forming osteoblasts [5].

The investigation of GP in CKD-induced growth retardation requires animal models because the analysis of GP is not feasible in children. The proximal GP of the tibiae of prepubertal rats has been used, although there is limited information on the alterations in the morphology and dynamics of GP and on the effect of CKD on the factors that regulate chondrocyte proliferation and differentiation. However, the chondrocytes of uremic rats are known to achieve a lower final size than those of control animals. This is crucial because the volume increase during chondrocyte hypertrophy is the main contributor to the longitudinal growth rate in mammals [6]. The increase in chondrocyte volume was found to result from three sequential stages: two stages of true hypertrophy (phases 1 and 3), when chondrocytes increase their volume in parallel with the active synthesis of cytoplasmic components, separated by a stage of swelling (phase 2), when the volume increases more than the synthesis, causing a decrease in the cytoplasm density. IGF1 was found to specifically affect chondrocyte hypertrophy during phase 3 [7].

We have recently developed a novel procedure that enables the objective discrimination of distinct groups of chondrocyte populations in the GP [8]. By applying this methodology to normal growing rats, we graded GP chondrocytes in seven different clusters, with four subphases in the pre-hypertrophic zone and three in the hypertrophic zone. In the present study, we apply this methodology to obtain new insights into the process of chondrocyte differentiation in a rat model of growth retardation induced by CKD. We also analyze the effects of GH treatment on the GP disturbances. For further molecular characterization of the alterations in the GP differentiation process in CKD, we analyze the immunohistochemical expression of the proteins associated with the process of chondrocyte hypertrophy.

## 2. Results

### 2.1. Renal Function and Mineral Metabolism

We used 3-week-old female rats which were grouped as follows: fed ad libitum with control diet (C); fed ad libitum with 0.5% adenine diet, either without treatment (AD) or with GH treatment (ADGH); and pair-fed with the AD group (PF). The AD and ADGH groups had similar degrees of renal failure, as shown by the similar serum creatinine and blood urea nitrogen (BUN) levels in Table 1. Phosphorus levels were significantly reduced in the AD, ADGH, and PF groups in comparison with the control group, but no significant differences in calcium levels were observed among all groups.

### 2.2. Growth Retardation and Uremic Growth Plate

As summarized in Table 2, AD animals were growth retarded, as demonstrated by their lower length gain and growth velocity, assessed by osseous front advance (OFA), compared with the PF and C groups. GH treatment normalized body weight, growth velocity, and food efficiency in ADGH rats.

**Figure 1:**
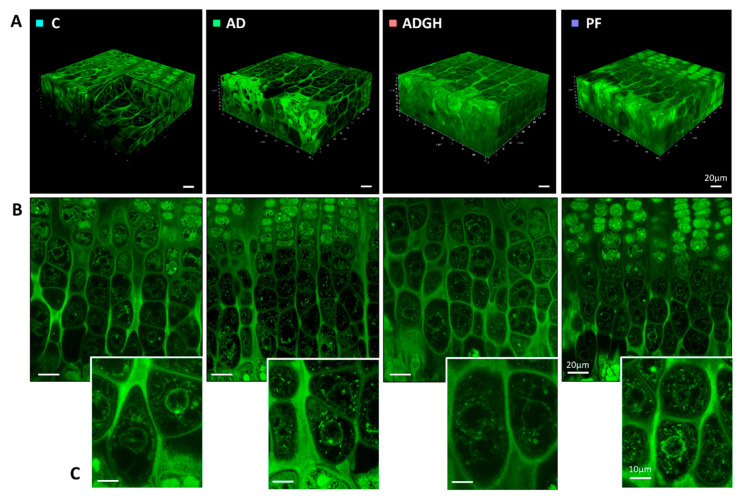
Confocal imaging of the growth plate cartilage. (**A**) A 3D projection of the thick section of the growth plate columns. Three-dimensional visualization is shown in Supplementary Videos 1–8. (**B**) Optical sections of focal planes of the hypertrophic zone. (**C**) Detail of terminal hypertrophic chondrocytes of the growth plates of the four experimental groups. C, control rats; AD, adenine 0.5% rats; ADGH, adenine 0.5% rats treated with growth hormone; PF, pair-fed rats.

When we examined the histology in the proximal metaphysis of the tibiae, the height of the GP was significantly smaller in the AD and PF groups than in the C group. The AD and PF groups also showed a decrease in the height of the hypertrophic zone compared with the C group (Table 3). GH treatment restored the micro-architecture of the GP (Figure 1) as well as both its total height and that of the hypertrophic zone (Table 3).

### 2.3. Three-Dimensional Analysis of Uremic Chondrocytes

**Figure 2:**
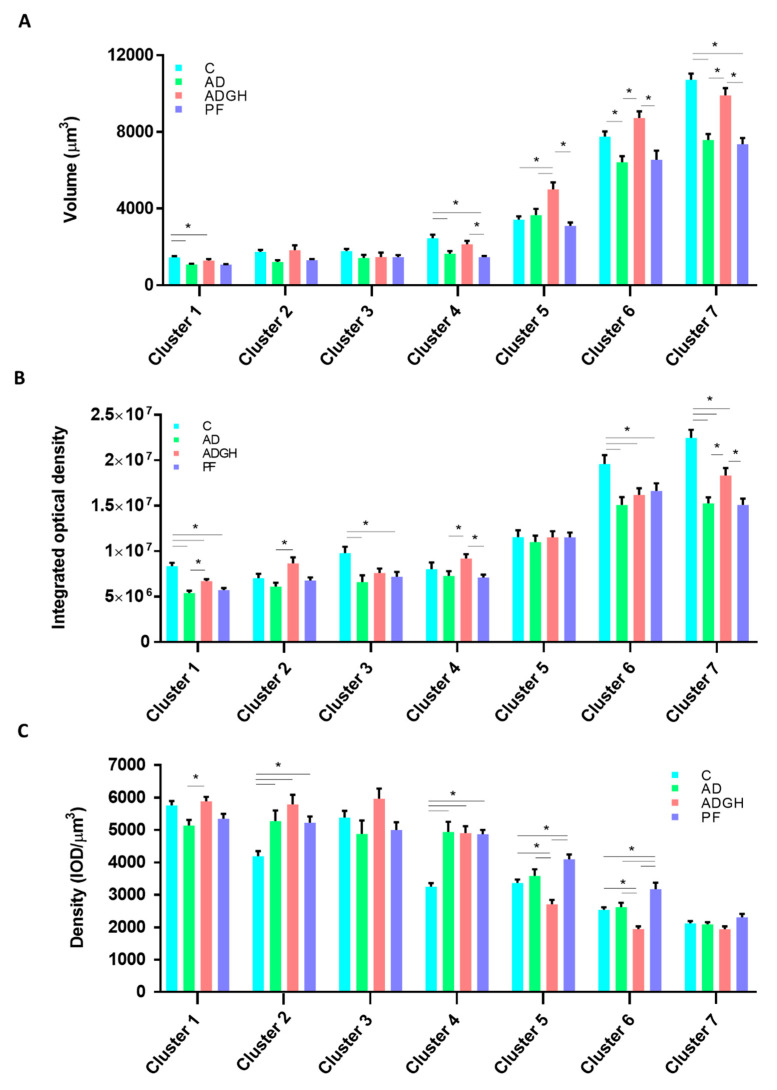
Quantitative values of chondrocytes of the four experimental groups. (**A**) Volume, (**B**) integrated optical density, and (**C**) cytoplasm density of chondrocytes of the groups organized into the seven categories of chondrocytes. Data obtained from a total of 1500 chondrocytes measured. C, control rats; AD, adenine 0.5% rats; ADGH, adenine 0.5% rats treated with GH; PF, pair-fed rats; IOD, integrated optical density. Values are mean ± SEM. * *p* value < 0.05.

In order to study how uremia and GH treatment affect the differentiation pattern of chondrocytes, we applied a three-dimensional approach, as described elsewhere [8]. Changes in the cell volume, integrated optical density (IOD), and cytoplasm cell density of the three-dimensional chondrocytes were analyzed along the long axis of the GP (Figure 1) (Videos S1–6). According to our results, the AD and PF groups started with a reduced chondrocyte cell volume in cluster 1, partially normalized from cluster 2 to 3 (Figure 2A,C). In the control GP, chondrocytes started enlarging in the transition from cluster 3 to 4. Conversely, AD chondrocytes were unable to increase their volume from cluster 3 to 4. By cluster 4, AD chondrocytes presented a significantly reduced volume and increased integrated optical density (IOD) compared to the control chondrocytes. The volume increase in the AD chondrocytes appeared to be delayed up to the transition from cluster 4 to 5, when they underwent an abrupt entrance in hypertrophy, by increasing their volume (×2.2) without a proportional increase in dry mass (×1.6). This progression differed greatly from that of the control chondrocytes, having a proportional increase in volume (×1.4) and dry mass (×1.4). Subsequently, in the transition from cluster 5 to 6, the control chondrocytes underwent a marked increase in their volume (×2.3) which was not accompanied by a proportional increase in dry mass. Instead, the AD chondrocytes had a more modest increase in volume and dry mass (×1.7 and ×1.30), causing a reduction in their cell cytoplasm density. Finally, in the transition from cluster 6 to 7, normal and uremic chondrocytes displayed parallel behaviors, undergoing both a proportional increase in volume and dry density, characteristic of true hypertrophy. Thus, uremic chondrocytes reached cluster 7 with a reduced size but at a normal cell density (Figure 1C and Figure 2A). The chondrocytes of PF animals mostly exhibited the same behavior as AD chondrocytes. They showed a similar pattern of volume and cell cytoplasm density from the prehypertrophic clusters 1 to 5 but a higher cell density from cluster 5 to 6, which was normalized in cluster 7 (Figure 2C).

The GP of the GH group (ADGH) showed a reduced chondrocyte volume in cluster 1, but the pattern of the chondrocyte volume increase was normalized from cluster 2 to 3. Chondrocytes increased their volume in the transition from cluster 3 to 4, and by cluster 4, they paralleled their volume with that of the control and significantly increased their IOD to higher values than the AD chondrocytes. In the transition from cluster 4 to 5, GH treatment caused a large volume enlargement (×2,3) which was not proportional to the increase in the IOD (×1,3), reducing the cell cytoplasm density even more than that of the AD chondrocytes. In the transitions from cluster 5 to 6 and 6 to 7, GH chondrocytes presented a similar pattern of increase in both volume (×1,7) and IOD (×1,3) than the uremic untreated chondrocytes (Figure 2A,B). By cluster 7, GH chondrocytes had a normalized chondrocyte volume (Figure 1C and Figure 2A).

### 2.4. Immunohistochemistry of Growth Plate Markers

**Figure 3:**
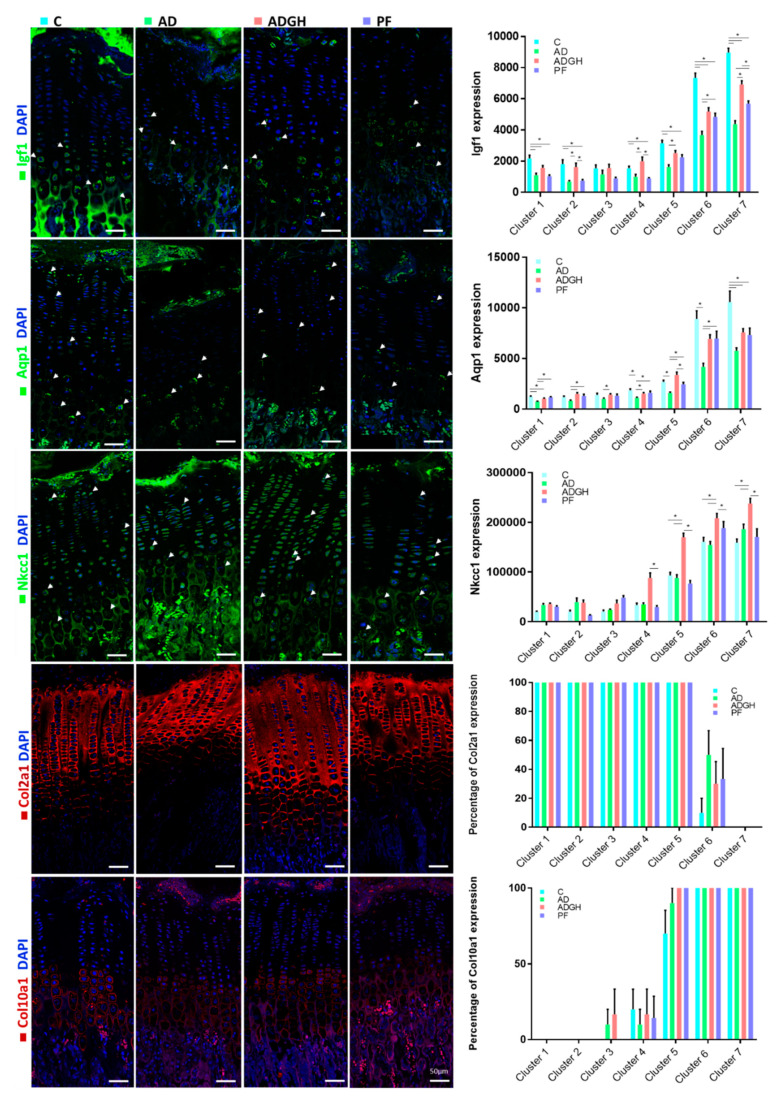
Immunohistochemical analysis of the expression of growth plate markers. Representative images are shown for Igf1 (green), Aqp1 (green), Nkcc1 (green), Col2a1 (red), and Col10a1 (red) and their corresponding quantifications in the seven described clusters. Arrowheads mark some of the labeled cells. Blue nuclei are stained by DAPI. DAPI, 4′,6-diamidino-2-phenylindole; C, control rats; AD, adenine 0.5% rats; ADGH, adenine 0.5% rats treated with GH; PF, pair-fed rats; Col2a1, collagen 2 alpha chain 1; Col10a1, collagen 10 alpha chain 1; Nkcc1, Na K Cl^−^ cotransporter 1; Aqp1, aquaporin 1; Igf1, insulin growth factor 1. Values are mean ± SEM. * *p* value < 0.05.

To connect the changes in volume, shape, and cytoplasm cell density in the chondrocyte subsets with changes in the specific proteins which are possibly implicated in the pathogenesis of growth impairment, we performed an immunohistochemical analysis (Figure 3). The pattern of the distribution of Igf1 was phenotypically similar between experimental groups, but its levels of expression were reduced in the AD group compared with the C group in all the GP clusters, except cluster 3, where levels did not differ between groups. In contrast, the PF group showed low levels, similar to those of AD, between clusters 1 to 5, but peaked in clusters 6 and 7, both with significantly more Igf1 expression than AD chondrocytes (Figure 3). GH treatment did not produce changes in the Igf1 levels between clusters 1 to 3 but increased its levels from clusters 4 to 6 compared with the AD group, to reach the highest levels in cluster 7 (Figure 3).

The expression of type II (Col2a1) and X (Col10a1) collagens was analyzed by inmunofluorescence (Figure 3). In control rats, Col2a1 expression was high in prehypertrophic clusters (clusters 1 to 5) and then showed a progressive decrease to reach relatively low values in the last clusters (clusters 6–7) (Figure 3). Col10a1 expression suddenly appeared in the early hypertrophy zone (cluster 4) and extended until late hypertrophy (cluster 7) (Figure 3). No apparent changes in the pattern of Col2a1 were found, but we did note an early Col10a1 expression in cluster 3, compared to the control (Figure 3).

The transporters and channels that regulate water and electrolyte traffic through the cell membrane Aquaporin (Aqp1) and Na^+^/K^+^/2Cl^−^ cotransporter 1 (Nkcc1) were analyzed. The Aqp1 signal was localized within the cytoplasm and the cellular membrane of the hypertrophic chondrocytes in all groups (Figure 3). Its expression was significantly diminished in the AD group compared to the C group for all the clusters. GH treatment appeared to partly normalize levels at clusters 2 to 6, but not at cluster 7, where levels were like those of uremic chondrocytes (Figure 3). Similarly, Nkcc1 expression was found in the hypertrophic chondrocytes in all groups, mainly in the cytoplasm and the cellular membrane (Figure 3). No significant changes were found in the pattern or intensity of expression in the AD and PF groups compared with the C group. Conversely, GH significantly increased Nkcc1 levels of expression from clusters 4 to 7 in comparison with the AD, PF, and C groups.

## 3. Discussion

In the present study, a three-dimensional approach to evaluate the alterations in the chondrocyte differentiation process that underlie growth retardation in CKD was applied for the first time. As previously described, the final chondrocyte volume was decreased in uremic rats, but GH treatment was able to normalize it. Interestingly, the pattern of variation in IOD and cell cytoplasm density suggests that uremia could be causing a delay at the beginning of chondrocyte hypertrophy. Nevertheless, GH treatment appeared to be able to compensate for this disturbance by triggering an early chondrocyte enlargement, likely mediated by Nkcc1 action (Figure 2 and Figure 3). For the experimental protocol, we used an adenine-induced CKD rat model that resulted in moderate renal failure, as reflected by BUN and serum creatinine concentrations that were about five times higher in the AD group than in the control and PF groups, and growth retardation, as previously described by our group [9]. As corresponds to the uremic state, the AD rats ate less than the ad libitum-fed rats with normal renal function (control group). It is of note that a similar degree of growth retardation was found in the AD and PF groups. In this model of uremia, a greater degree of growth impairment in the AD rats could have been achieved by a higher dose of adenine in their diets, as adenine is not well tolerated by rats and causes more severe bone diseases than in rats with subtotal nephrectomy [10].

The impairment of growth was associated with several modifications in the morphology and dynamics of the GP, as previously reported [11,12]. The height of the growth plate was significantly reduced in uremic and PF rats. It has been indistinctly described to be increased, reduced, or unchanged compared to the controls, a fact suggesting that it could be dependent to a great extent on the severity of the uremia [13]. AD rats presented smaller GPs, and this reduction was accompanied by a significantly reduced hypertrophic stratum (Table 3). Previous studies are coincident with these findings and also show that the GP height reduction is caused by alterations in the hypertrophic zone rather than by modifications in the proliferative activity, although this is also reduced in CKD [9,14].

The hypertrophy of GP chondrocytes is known to be the main contributor to bone lengthening [6]. The hypertrophic chondrocytes of uremic rats have been reported to achieve a lower final size than those of control animals [12]. This process is characterized by a widespread increase in cell volume and has been studied previously, especially with respect to its regulation [15]. However, information regarding how the cell volume increase occurs is limited. The increase in chondrocyte volume has been reported to result from three sequential phases, as mentioned above [7,8]. By applying our technique to the chemically fixed, in situ chondrocytes of uremic rats, we demonstrated here that the chondrocytes of uremic animals started with a reduced chondrocyte volume and cell density, as seen in cluster 1. The GP chondrocytes of normal growing rats started enlarging in the transition from cluster 3 to 4, when the beginning of hypertrophy occurs. Conversely, by cluster 4, uremic chondrocytes seemed unable to start increasing and volume enlargement appeared to be delayed until the transition from cluster 4 to 5. Our results suggest that uremic chondrocytes, by cluster 4, had the same amount of proteins as normal chondrocytes (Figure 2B), but, somehow, they were unable to use this to start enlarging, causing hypertrophy to be delayed. Interestingly, the start of the hypertrophy was quite abrupt, as seen in the transition from cluster 4 to 5 and 5 to 6, when they underwent two phases of marked volume increase, causing the cell cytoplasm density to decay, compared to the control (Figure 2A,C). At a later stage of hypertrophy, the behavior of normal and uremic chondrocytes became parallel, both experiencing a phase characteristic of true hypertrophy. These results suggest that, in CKD, chondrocytes are not able to reach a normal terminal chondrocyte volume because they experience a delayed start to hypertrophy and seem unable to catch up, reaching the end of the maturation process with a reduced size. The chondrocytes of PF animals mostly show the same behavior as uremic chondrocytes, as suggested by the cell volume pattern across all the GP clusters. This could suggest that some of the GP alterations during CKD are not caused by uremia itself but are partially triggered by associated nutritional disorders. More marked differences between the chondrocytes of PF and AD rats had likely been found by inducing a more severe degree of renal failure [13]. However, in this model of uremia, this would have required a higher concentration of adenine in the diet, which is not well tolerated by rats and induces life-threatening inflammation [9].

Studies in mice lacking the GH receptor, Igf1, or both, provided conclusive evidence suggesting that only 17% of postnatal growth occurs independent of the GH/IGF-1 axis [16]. Growth impairment in CKD children has been explained mainly by a reduced IGF-I bioavailability, in part owing to a GH-resistance state caused by a postreceptorial defect in the JAK2-STAT5b signaling pathway downstream of the GH, as shown in the liver, skeletal muscle, and GP [3,17,18,19,20]. Our previous findings revealed low Igf1 expression levels in the prehypertrophic clusters and a significant increase in the transition from cluster 4 to cluster 5, coincident with the onset of chondrocyte hypertrophy in the transition from cluster 5 to cluster 6 and in the transition from cluster 6 to cluster 7, to reach the highest expression level at the later stage of the hypertrophy process [8]. In uremic chondrocytes, this pattern of expression was preserved, but there were significantly reduced levels of expression across the GP, more importantly in the hypertrophic clusters. These results are in line with those previously seen by Troib et al. [21], which showed reduced protein expression, suggesting that a decrease in the action of GH, mediated through the JAK2/STAT5 pathway in the bone during CKD, in turn, leads to a decreased local Igf1 level. GP Igf1 expression was reduced in the food-restricted, pair-fed, normal control rats compared with the ad libitum-fed, normal rats. This again indicated that the suppressed GP Igf1 expression in CKD is in part secondary to anorexia and reduced nutrient intake. Reduced levels of Igf1 mRNA and protein were associated with chronic anorexic states or prolonged fasting or food restriction [20,21,22]. Gevers et al. [23] demonstrated that fasting attenuates the Stat5 phosphorylation response to GH therapy in the liver and the GP cartilage of GH-deficient mice, which could partly explain the similar behavior of uremic and pair-fed animal chondrocytes.

Other factors besides GH and IGF-I contribute to the rate of bone growth through endochondral ossification, including the control of chondrocyte cell volume and proliferation rates. Type II and type X collagen are the most abundant proteins found in the GP. Type II is the predominant structural collagen present in all zones of the GP and is the main protein that is responsible for providing the tissue with a fibrillar framework to arrange chondrocytes as well as other extracellular matrix components (ECM). Type X collagen is an ECM component expressed exclusively by hypertrophic chondrocytes. No changes were observed in the pattern of expression of Col2a1 in uremic GPs, but we did note an early Col10a1 expression, by cluster 3. Interestingly, previous studies also revealed a generalized decrease in collagen expression and changes in its architecture, which were especially marked in the hypertrophic cartilage of 5/6 nephrectomized rats [24].

The distribution of the transporters and channels that regulate water and electrolyte traffic through the cell membrane was also analyzed, given its importance in the cell enlargement characteristic of hypertrophy. Our aim was to evaluate if some proteins could be implicated in the pathogenesis of growth retardation in uremia. Water channels facilitate plasma membrane water permeability to the levels required for efficient coupling between NaCl transport and water transport in epithelia, which carries out isosmotic fluid transport [25]. Aqp1 is a widely expressed water channel whose physiological function has been most thoroughly characterized in the kidney, but it has also been found in red blood cells, the vascular endothelium, the gastrointestinal tract, sweat glands, and lungs [26]. Aqp1 expression was recently described in GP chondrocytes, mostly in the hypertrophic zone. No differences in the pattern of distribution of Aqp1 were reported in uremia or when treated with GH [12]. Our results confirmed these findings and showed that there are indeed differences in the levels of expression and that Aqp1 levels are significantly diminished in uremia in almost all the GP strata. Alternatively, we have found that uremia does not seem to alter the levels of expression of Nkcc1, an important membrane cotransporter in the GP chondrocyte enlargement [27]. There is no precise knowledge of the actions of either Nkcc1 or Aqp1 in the hypertrophy of chondrocytes, so further experiments would be required in order to unravel their mechanism of action in the cell volume enlargement.

High pharmacological doses of GH accelerate growth velocity [28] and improve the final height of patients with CKD [29]. The effects of GH therapy in the GP chondrocytes of uremic rats have not been extensively studied. The present study found that GH normalizes the growth rate, measured by OFA, and the height of both the GP and the hypertrophic stratum. Remarkably, the three-dimensional study of the uremic chondrocytes treated with GH revealed that, whereas chondrocytes, like untreated chondrocytes, start with a reduced chondrocyte volume, the pattern of chondrocyte volume increase is partly normalized at the prehypertrophic clusters compared to the control. GH appeared to be able to successfully enable uremic chondrocytes to enter hypertrophy, and in cluster 4, they present normal volumes. In the remaining phases, GH chondrocytes underwent the same volume enlargement as the untreated uremic chondrocytes, but, coming from a higher volume, they reached cluster 7 with a significantly increased volume. Even if GH was not able to normalize the pattern of hypertrophy in uremic chondrocytes from clusters 5 to 7, it was able to stimulate a chondrocyte cell volume enlargement from clusters 3 to 5. These data are consistent with the previous findings stating that GH acts locally at the GP to recruit resting chondrocytes into a proliferative state [30], as well as to stimulate local Igf1 production, which then stimulates the proliferation of chondrocytes [4,30,31]. In turn, our data suggest that GH treatment stimulates protein synthesis at the prehypertrophic stages of chondrocytes, causing an early increase in the volume of chondrocytes and allows them to complete the maturation process with normal parameters of volume and cytoplasm cell density. Moreover, GH increases Igf1 levels. Thus, Igf1 may play a role in the GH’s compensatory mechanism of chondrocyte volume increase. Findings in experimental animals and in humans have shown that the absence or mutations of the STAT5b gene, the major mediator of the GH-regulated IGF-1 gene expression, are associated with diminished postnatal growth, GH resistance, and reduced IGF-I synthesis [32].

In this study, GH was able to raise Aqp1 levels at the prehypertrophic stages and allow chondrocytes to enter hypertrophy with significantly increased levels compared to uremic chondrocytes (cluster 4 to 5), a result that differs from previously published studies [10]. The level of Aqp1 expression in GH-treated chondrocytes, even if significantly lower than the control in most of the GP, has the same pattern of variation across the GP than untreated chondrocytes, indicating that, even though Aqp1 is still functioning, it could not be a part of the mechanism by which GH normalizes the chondrocyte volume in uremic conditions. However, it is interesting to note that, whereas uremic chondrocytes maintain normal Nkcc1 levels, GH causes an early Nkcc1 expression by cluster 4, even when compared to the control. In a recent study, we proposed that, in normal growing rats, Nkcc1 may have a major role in the onset of cell volume expansion [8]. Treatment with pharmacologic doses of GH has been shown to overcome the GH-resistance state and be effective in normalizing STAT5 phosphorylation [12,33]. Thus, it is tempting to propose that GH treatment could increase Nkcc1 levels and so increase chondrocyte volume at an early stage. Further studies in this line of work would be of great importance.

## 4. Materials and Methods

### 4.1. Animals

The study was carried out in three-weeks-old female Sprague–Dawley rats (Charles River Laboratories, L’Arbrelese, France). The procedures involving animals and their care were conducted according to Spanish law on the use of experimental animals, which acknowledges the European Directive 86/609. The project proposal was approved by the Ethical Committee of the University of Oviedo, Spain (14 July 2015, PROAE 19/2015).

Rats were housed in light (12 light/dark cycles) and temperature-controlled rooms (21–23 °C). Diets were purchased from Ssniff Spezialdiäten GmbH (ref V1534. Soest, Germany). All animals had free access to tap water. Four groups of 10 animals were used: C (rats fed with normal diet), AD (rats fed with 0.5% adenine diet), ADGH (AD rats treated with GH), and PF (rats fed with normal diet, pair-fed with AD group). Recombinant human GH (rhGH) (Norditropin^®^), gently provided by Novo Nordisk Pharma, Madrid, Spain), was administered intraperitoneally to the ADGH group from day 14 to day 20 of the protocol, at a dosage of 3.3 mg/kg/day, given at 09.00 h and 17.00 h [34]. The other groups received the vehicle following an identical protocol of administration. Animals were sacrificed under a lethal dose of Dolethal^®^ anesthesia after 21 days. Bromodeoxyuridine (BrdU) (100 mg/kg; Sigma Aldrich, Madrid, Spain) was injected intraperitoneally 1 and 8 h before sacrifice. Five days before sacrifice, 20 mg/kg of intraperitoneal calcein (Sigma, St Louis, MO, USA) was injected for the measurement of the OFA.

### 4.2. Blood Biochemistry

Serum concentrations of creatinine, urea nitrogen, calcium, and phosphate were measured by IDEXX laboratories (Barcelona, Spain).

### 4.3. Growth and Nutrition

Growth during the experimental protocol was assessed by measuring the total gained weight between days 0 and 21. Animals were weighed daily using a scale. Likewise, the length from the snout to the distal end of the tail was measured under anesthesia on day 21 of the protocol using a millimeter rule. Longitudinal growth rate (μm/day) was assessed by OFA in frontal sections from the right tibias. Sections were examined under an Olympus incident light fluorescent microscope (Olympus BX61, Olympus Optical, Barcelona, Spain) to detect calcein labels. Images were captured and measurements were made using Image J (National Institutes of Health, Bethesda, Maryland, MD, USA). The mean value measurement divided by 5 days was considered the osseous front advance per day, representing the daily longitudinal bone growth rate in each animal.

Food efficiency was calculated as the ratio between weight gained and food consumed (*g*/*g*) by each animal between days 0 and 24 of the protocol.

### 4.4. Sample Harvest and Processing

The rats’ right tibias were fixated in glutaraldehyde 2%, ruthenium hexaammine trichloride 0.5%, and calcium chloride 5 mM in 0.025 M sodium cacodylate buffer (pH 7.4, osmolarity 300 mOsm). Then, they were in-block stained with a solution of 0.5% eosin in acetate buffered ethanol for 2 h at 4 °C, after which they were embedded in epoxy resin Durcupan (Sigma) and thinned by grinding to obtain 100-μm thick bone sections, as previously described by our group for the three-dimensional analysis of the GP [8].

The left tibias, fixed in 4% paraformaldehyde (PFA) and embedded in methyl methacrylate [35], were used for the histological and inmunofluorescence analyses.

### 4.5. Three-Dimensional Reconstruction of the Growth Plate

The bone thick sections were imaged with a confocal microscope Leica TCS SP8 (Leica Microsystems, Germany) equipped with a pulsed white light laser (470–670 nm), Acousto-Optical Beam Splitter (AOBS), and two internal hybrid single photon counting detectors, which was operated by Leica Application Suite X program (Leica Microsystems, Wetzlar, Germany). Z-stacks of 184.52 μm (x) × 184.52 μm (y) × 50 μm (z) with an X/Y resolution of 1024 × 1024 pixels were obtained by the sequential overlapping of 100 continuous images at an increment of Z-axis optical section of 0.5 μm. Two scans were performed along the XY plane, sequential in the Y axis, to obtain an image reconstruction of a complete column; see Supplementary Videos S1–6. The Leica LAS X 3D software was used for the 3D projection and the IMARIS v. 7.1.1. software (Bitplane, Switzerland) to obtain the cell structural parameters of the chondrocytes: cell volume, integrated optical density (IOD), and sphericity. The sequence of structural variation along a complete column was analyzed by measuring a total of 300 chondrocytes for each experimental group.

### 4.6. Immunohistochemistry and Immunofluorescence Analysis

The fluorescent immunodetection of Col2a1, Col10a1, Nkcc1, Aqp1, and Igf1was performed in 5 µm thick sections of methyl-methacrylate embedded left tibias. Antigen retrieval was performed with proteinase K 1 mg/mL 16 min at 37 °C for Igf1, hyaluronidase 2 mg/mL 30 min 37 °C for Nkcc1, and citrate buffer pH 8 30 min at 60 °C for Aqp1. A TNB blocking buffer made with 0.1 M TRIS-HCl, pH 7.5 0.15 M NaCl 0.5% TSA Blocking Reagent (FP1012, Perkin Elmer, Waltham, Massachusetts, MA, USA) was used. Antigen retrieval was performed with proteinase K 1 mg/mL 15 min at 37 °C for Col2a1, proteinase K 1 mg/mL 10 min at 37 °C for Col10a1, proteinase K 1 mg/mL 16 min at 37 °C for Igf-1, hyaluronidase 2 mg/mL 30 min 37 °C for Nkcc1, and citrate buffer pH 8 30 min at 60 °C for Aqp1. A TNB blocking buffer (75 min, RT) made from a TSA blocking reagent (Perkin Elmer, Waltham, MA, USA) was used. The primary antibodies used were as follows: anti-Col2a1 (#MA5-12789, Invitrogen, California, CA, USA) at 1:20 dilution, anti-Col10a1 (A6889, Abclonal, MA 01801, USA) at 1:20 dilution, anti-Igf1 (#PA5-27207, Invitrogen, California, CA, USA) at 1:200 dilution, anti-Aqp1 (ab15080, Abcam, Cambridge, UK) at 1:100 dilution, and anti-Nkcc1 (ab59791, Abcam, Cambridge, UK) at 1:100 dilution. Goat anti-mouse Alexa 594 (A-21155, Invitrogen, California, CA, USA), goat anti-rabbit Alexa 594 (#A-11012, Invitrogen, California, CA, USA), and goat anti-rabbit Alexa 488 (A11034, Invitrogen, California, CA, USA) were used as secondary antibodies, and sections were finally mounted with Vectashield Mounting Medium with DAPI (Vector Laboratories, Burlingame, California, CA, USA). Sections were examined and pictures taken with a confocal microscope, the Leica TCS SP8 (Leica Microsystems, Wetzlar, Germany), with the 20× magnification objective. Image J (National Institutes of Health, Bethesda, Maryland, MD, USA) was used to measure the fluorescence intensity on the whole chondrocyte columns.

Sections were examined and pictures taken with a confocal microscope, Leica TCS SP8 (Leica Microsystems, Germany), with the 20× magnification objective. Image J (National Institutes of Health, Bethesda, Maryland, MD, USA) was used to measure the fluorescence intensity on the whole chondrocyte columns and the position of chondrocytes in the GP.

### 4.7. Statistical Analysis

All statistical analyses utilized a 95% confidence level and were conducted using GraphPad Prism v. 7 (La Jolla, California, CA, USA).

To analyze the structural variation along the vertical column, chondrocytes were classified into the previously described differentiation clusters [8], based on their distance to the osseous invasion front. Means and standard deviations (SD) of the different structural parameters were determined for each cluster. Comparison among experimental groups was performed using a one-way ANOVA, followed by Tukey’s multiple comparison test.

## 5. Conclusions

By applying for the first time a three-dimensional approach to study the abnormalities in the GP maturation of uremic chondrocytes, we have seen that uremic chondrocytes experience a delayed entrance into hypertrophy, preventing them from reaching a normal size by the end of the differentiation process. Growth hormone treatment appears to be able to compensate for this by triggering an early chondrocyte enlargement that may be somehow mediated by Nkcc1 action.

## Figures and Tables

**Table 1:** Serum biochemical determinations in the four groups of rats (*n* ≥ 5 per group).

Groups	Serum Creatinine (mg/dL)	BUN (mg/dL)	Calcium (mg/dL)	Phosphorus (mg/dL)
C	0.12 ± 0.01	23d ± 2	9.16 ± 0.22	10.16 ± 0.27
AD	0.59 ± 0.05 ^a^	122 ± 8 ^a^	9.45 ± 0.15	8.00 ± 0.47 ^a^
ADGH	0.51 ± 0.06 ^a^	92 ± 9 ^a,b^	9.16 ± 0.23	8.33 ± 0.44 ^a^
PF	0.10 ± 0.01 ^b,c^	40 ± 2 ^b,c^	8.96 ± 0.09	6.79 ± 0.25 ^a,c^

BUN, blood urea nitrogen; AD, 0.5% adenine diet; ADGH, 0.5% adenine diet and growth hormone (GH) treatment; C, normal diet; PF, normal diet pair-fed with the AD group. Values are mean ± SEM. ^a^ Compared with C group, *p* < 0.05. ^b^ Compared with AD group, *p* < 0.05. ^c^ Compared with ADGH group, *p* < 0.05.

**Table 2:** Growth parameters in the groups of rats (*n* ≥ 5 per group).

Groups	Nose–Tail Length Gain (cm)	Body Weight Gain (g)	Food Efficiency (*g*/*g*) *	OFA (µm/Day)
C	9.10 ± 0.28	107.30 ± 5.04	0.29 ± 0.01	335.7 ± 10.25
AD	4.49 ± 0.31 ^a^	54.12 ± 2.59 ^a^	0.23 ± 0.01 ^a^	210.3 ± 6.33 ^a^
ADGH	5.01 ± 0.44 ^a^	68.53 ± 4.71 ^a,b^	0.28 ± 0.01 ^b^	288.7 ± 17.97 ^b^
PF	4.96 ± 0.22 ^a^	43.28 ± 2.57 ^a,c^	0.18 ± 0.01 ^a,b,c^	206.2 ± 16.07 ^a,c^

AD, 0.5% adenine diet; ADGH, 0.5% adenine diet and GH treatment; C, normal diet; PF, normal diet pair-fed with the AD group; OFA, osseous front advance. Values are mean ± SEM. * Food efficiency was calculated as grams of gained weight divided by grams of consumed food; ^a^ Compared with C group, *p* < 0.05. ^b^ Compared with AD group, *p* < 0.05. ^c^ Compared with ADGH group, *p* < 0.0.5.

**Table 3:** Histological characteristics of the proximal tibial growth plate in the four groups of rats (*n* ≥ 5 animals per group).

Groups	GP Height (μm)	HZ Height (μm)
C	384.5 ± 12.42	173.8 ± 10.79
AD	323.6 ± 12.47 ^a^	132.4 ± 6.016 ^a^
ADGH	416.2 ± 30.82 ^b^	166.2 ± 22.18
PF	281.7 ± 9.78 ^a,c^	109.3 ± 6.04 ^a,c^

AD, 0.5% adenine diet; ADGH, 0.5% adenine diet and GH treatment; C, normal diet; PF, normal diet pair-fed with the AD group; GP, growth plate; HZ, hypertrophic zone. Values are mean ± SEM. ^a^ Compared with C group, *p* < 0.05. ^b^ Compared with AD group, *p* < 0.05. ^c^ Compared with ADGH group, *p* < 0.05.

## Supplementary Materials

Supplementary materials can be found at https://www.mdpi.com/1422-0067/21/12/4519/s1. Video S1. Representative video of a three-dimensional-reconstruction of Z-series confocal images of a thick bone section of an AD rat proliferative zone imaged with the confocal microscope. Travel through xz-projections of a confocal z-stack; Video S2. Representative video of a three-dimensional-reconstruction of Z-series confocal images of a thick bone section of an AD rat hypertrophic zone imaged with the confocal microscope. Travel through xz-projections of a confocal z-stack; Video S3. Representative video of a three-dimensional-reconstruction of Z-series confocal images of a thick bone section of an ADGH rat proliferative zone imaged with the confocal microscope. Travel through xz-projections of a confocal z-stack; Video S4. Representative video of a three-dimensional-reconstruction of Z-series confocal images of a thick bone section of an ADGH rat hypertrophic zone imaged with the confocal microscope. Travel through xz-projections of a confocal z-stack; Video S5. Representative video of a three-dimensional-reconstruction of Z-series confocal images of a thick bone section of an PF rat proliferative zone imaged with the confocal microscope. Travel through xz-projections of a confocal z-stack; Video S6. Representative video of a three-dimensional-reconstruction of Z-series confocal images of a thick bone section of an PF rat hypertrophic zone imaged with the confocal microscope. Travel through xz-projections of a confocal z-stack.

## Abbreviations

BUN	Blood urea nitrogen
CKD	Chronic kidney disease
DAPI	4′,6-diamidino-2-phenylindole
ECM	Extracellular matrix
GP	Growth plate
GH	Growth hormone
Igf1	Insulin growth factor 1
IOD	Integrated optical density
BrdU	Bromodeoxyuridine
Aqp1	Aquaporin 1
Nkcc1	Sodium potassium calcium cotransporter 1
Col2a1	Collagen 2 alpha chain 1
Col10a1	Collagen 10 alpha chain 1
