# Supplementary material for: Innovative Three-Dimensional Microscopic Analysis of Uremic Growth Plate Discloses Alterations in the Process of Chondrocyte Hypertrophy: Effects of Growth Hormone Treatment
Source: Int J Mol Sci. 2020 Jun 25;21(12):4519. doi: 10.3390/ijms21124519 (PMC7350242; doi:10.3390/ijms21124519)
Supplement: Supplementary file 1 [file ijms-21-04519-s001.zip › 02_Supplementary data legends.docx]

**Title: Innovative three-dimensional microscopic analysis of uremic growth plate discloses alterations in the process of chondrocyte hypertrophy. Effect of growth hormone treatment**

Authors: Ángela Fernández-Iglesias^1,2^, Rocío Fuente^1^, Helena Gil-Peña^1,2,4^, Laura Alonso-Durán^1,2^, María García-Bengoa^1^, José Manuel López^1,3^ and Fernando Santos^1,2,4^

**Supplementary data**

*Legends for supplementary videos*

Video S1.

Representative video of a three-dimensional-reconstruction of Z-series confocal images of a thick bone section of an AD rat proliferative zone imaged with the confocal microscope. Video was made via the “Movie Maker” function with the increase in display time in association with the depth of the optical section. Travel through xz-projections of a confocal z-stack.

Video S2.

Representative video of a three-dimensional-reconstruction of Z-series confocal images of a thick bone section of an AD rat hypertrophic zone imaged with the confocal microscope. Video was made via the “Movie Maker” function with the increase in display time in association with the depth of the optical section. Travel through xz-projections of a confocal z-stack.

Video S3.

Representative video of a three-dimensional-reconstruction of Z-series confocal images of a thick bone section of an ADGH rat proliferative zone imaged with the confocal microscope. Video was made via the “Movie Maker” function with the increase in display time in association with the depth of the optical section. Travel through xz-projections of a confocal z-stack.

Video S4.

Representative video of a three-dimensional-reconstruction of Z-series confocal images of a thick bone section of an ADGH rat hypertrophic zone imaged with the confocal microscope. Video was made via the “Movie Maker” function with the increase in display time in association with the depth of the optical section. Travel through xz-projections of a confocal z-stack.

Video S5.

Representative video of a three-dimensional-reconstruction of Z-series confocal images of a thick bone section of an PF rat proliferative zone imaged with the confocal microscope. Video was made via the “Movie Maker” function with the increase in display time in association with the depth of the optical section. Travel through xz-projections of a confocal z-stack.

Video S6.

Representative video of a three-dimensional-reconstruction of Z-series confocal images of a thick bone section of an PF rat hypertrophic zone imaged with the confocal microscope. Video was made via the “Movie Maker” function with the increase in display time in association with the depth of the optical section. Travel through xz-projections of a confocal z-stack.
